# Quantum-Dot-Based Molecularly Imprinted Hydrogel for Rapid Detection of Homocysteine

**DOI:** 10.3390/gels11080632

**Published:** 2025-08-11

**Authors:** Xin Zhang, Jiarong Liang, Binglei Zheng, Pengfei Jiao, Qian Xu

**Affiliations:** Research Center of Henan Provincial Agricultural Biomass Resource Engineering and Technology, College of Life Science, Nanyang Normal University, Nanyang 473061, China; 13620667641@163.com (J.L.); 13459281791@163.com (B.Z.); jiaopf@nynu.edu.cn (P.J.)

**Keywords:** quantum dots, molecularly imprinted hydrogel, homocysteine, fluorescence sensor

## Abstract

Elevated levels of homocysteine (Hcy) are associated with various pathological conditions including atherosclerosis, hypertension, and cardiovascular diseases. In this work, quantum-dot-based molecularly imprinted hydrogels (QD@MIHs) were developed by integrating L-cysteine-modified ZnS quantum dots (QDs)with highly selective molecular imprinting technology for rapid homocysteine detection. The QD@MIPHs were fabricated using a dual-functional monomer system (acrylamide and methacrylic acid) through surface coating of the Hcy molecularly imprinted polymer gel onto the QDs. Under optimal conditions, the response time of the QD@MIPHs for Hcy detection was 5 min. When the Hcy concentration ranged from 0.1 to 10.0 μM, the fluorescence quenching of the QD@MIHs showed a good linear relationship with Hcy concentration (*R*^2^ = 0.9972), with a corresponding detection limit of 0.027 μM. In addition, the constructed QD@MIPHs showed no significant response to other interfering substances, demonstrating the high selectivity of the prepared material. Practical sample analysis revealed that the recovery rates of Hcy ranged from 94.34% to 104.1%, with relative standard deviations (RSD, *n* = 3) between 3.56% and 7.17%. This study provides a novel tool and method for rapid Hcy detection with significant potential in biomedical diagnostics and preventive-healthcare applications.

## 1. Introduction

Homocysteine (Hcy) is a crucial intermediate metabolite in the methionine and cysteine metabolic pathways in humans [1]. Under physiological conditions, serum homocysteine is metabolized via enzymatic reactions into glutathione and *S*-adenosylmethionine, participating in protein biosynthesis and cellular metabolism maintenance, thereby playing a pivotal role in both metabolic regulation and immune function [2,3,4]. However, when this metabolic process is disrupted by factors such as genetic predisposition, nutritional status, unhealthy lifestyle, aging, chronic diseases, or certain medications, Hcy and its metabolites accumulate in the blood, leading to elevated homocysteine levels (hyperhomocysteinemia) [5,6,7,8]. Studies have shown that a variety of diseases such as stroke, depression, Alzheimer’s disease, cardiovascular disease, certain cancers and gestational hypertension are associated with high levels of Hcy in the blood [9,10,11,12,13]. Therefore, the detection of Hcy has received extensive attention in the fields of drug research, medical diagnosis and disease monitoring. Currently, there are numerous detection methods for detecting homocysteine, including enzyme-linked immunosorbent assay (ELISA), gas chromatography-mass spectrometry (GC-MS), high-performance liquid chromatography (HPLC), capillary electrophoresis, and fluorescence polarization immunoanalysis [14,15,16,17,18,19,20,21]. Although these techniques can provide reliable Hcy detection results, they still present several limitations, such as complex pretreatment, high operational costs, and the requirement for specialized instruments and trained personnel. Therefore, it is highly necessary to develop a faster, more convenient, and lower-detection-limit Hcy determination technology. Due to their advantages such as low cost, reliability, excellent selectivity, high sensitivity, and portability, molecularly imprinted sensors have become a promising candidate for the development of novel Hcy detectors.

Molecular imprinting technology (MIT) is a well-established technique that involves copolymerization and cross-linking of functional monomers with template molecules, followed by template removal to create specific recognition sites [22,23,24]. Molecularly imprinted hydrogels (MIHs) are a novel functional material that combines MIT with hydrogel matrices [25]. By incorporating specific molecular recognition sites into the hydrogel network, MIHs not only retain the high water content, swelling capacity, and biocompatibility of conventional hydrogels but also exhibit highly selective and specific adsorption capabilities for target molecules [26,27,28]. Due to their intelligent molecular recognition and responsive properties, MIHs demonstrate broad application prospects in biomedical fields, food, and industrial separation [29,30,31,32,33]. However, during conventional MIH preparation, template molecules tend to become entrapped or embedded within the gel network, leading to difficult template removal and mass transfer limitations. These issues adversely affect the adsorption performance and specificity of MIHs, significantly hindering their practical applications.

Quantum dots (QDs) hold significant application value in sensor development due to their exceptional physical and optical properties, such as narrow emission spectra, high quantum yields, and size-tunable fluorescence characteristics [34,35,36]. The unique properties of QDs enable the development of highly sensitive sensing platforms. The novel intelligent sensing platform constructed by integrating QDs with molecularly imprinted hydrogels combines the superior signal transduction of QDs with the specific molecular recognition advantages of MIHs [37,38,39]. The molecularly imprinted hydrogel not only effectively protects quantum dots (QDs) from environmental quenching and enhances their stability, but its environmental responsiveness (such as pH and temperature) also enables intelligent regulation of detection conditions [40,41]. Furthermore, the molecularly imprinted cavities within the hydrogel can specifically capture target molecules, significantly improving detection selectivity.

In this study, quantum dots were integrated with molecularly imprinted hydrogels to fabricate a QD-based MIH fluorescence sensor for the detection of homocysteine, employing acrylamide and methacrylic acid as dual-functional monomers. The QD@MIPHs not only retain the conventional advantages of molecularly imprinted polymers, including excellent physical stability, high selectivity, low cost, and broad operating conditions, but also demonstrate additional merits such as a fast mass transfer rate, easy elution/adsorption, and a low detection limit. The developed sensor exhibits high sensitivity and accuracy in Hcy quantification, enabling reliable clinical assessment of cardiovascular disease risk and facilitating early intervention. Furthermore, the QD@MIH system demonstrates broad applicability in biomarker detection, environmental monitoring, and food safety analysis, offering enhanced sensitivity and specificity while paving the way for portable, real-time sensing devices.

## 2. Results and Discussion

### 2.1. Fabrication of Hcy Molecularly Imprinted Hydrogel

**Figure 1:**
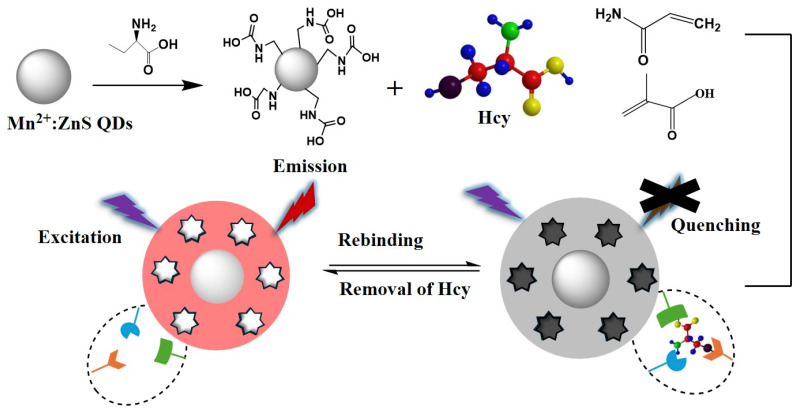
Schematic of the QD@MIHs preparation process.

QD@MIHs were prepared through surface molecular imprinting technology by copolymerizing dual-functional monomers (AAM and MAA) on *L*-cysteine-modified Mn^2+^-doped ZnS quantum dots to construct a molecularly imprinted hydrogel system. The specific preparation process is illustrated in Figure 1. First, water-soluble manganese-doped ZnS quantum dots (QDs) were employed as the signal transduction unit and substrate nanomaterial. ZnS QDs exhibit narrow emission bands, and doping with Mn^2+^ can further enhance their fluorescence quantum yield and photochemical stability. Meanwhile, the ZnS QDs were surface-modified with L-cysteine to improve their biocompatibility while enhancing their optical stability and aqueous dispersibility. Second, AAm and MAA were selected as dual-functional monomers to construct a molecularly imprinted hydrogel layer for Hcy on L-cysteine-modified ZnS quantum dots via surface imprinting technology. MAA provided pH sensitivity to the imprinted gel while AAm regulated the gel network elasticity and swelling rate by harnessing their synergistic effects. Finally, after eluting the Hcy molecules, complementary binding sites matching Hcy in terms of structure, size, shape, and functional groups were formed in the molecularly imprinted hydrogel on the prepared QD@MIHs. The constructed molecularly imprinted hydrogel coating on the QDs surface not only provided selectivity for Hcy but also prevented other interfering substances from binding to the QD@MIHs surface. Furthermore, the constructed QD@MIH exhibited pH-responsive characteristics, enabling rapid binding of Hcy molecules in samples through pH adjustment. In this study, QD@MIHs exhibited characteristic orange-red fluorescence emission at 594 nm in the absence of homocysteine (Hcy). Upon Hcy addition, the molecularly imprinted hydrogel layer on the QD@MIHs surfaces selectively captured Hcy molecules through specific binding interactions, leading to significant fluorescence quenching. Stern-Volmer analysis confirmed that the quenching efficiency showed a positive linear correlation with Hcy concentration. The fluorescence quenching mechanism involves photoinduced electron transfer from the excited state of quantum dots to Hcy molecules, introducing a new nonradioactive decay pathway for the excited-state energy [42]. This dynamic quenching process ultimately converts the molecular recognition event into analyzable optical signals, enabling rapid detection of the target molecule in the samples.

### 2.2. Characterization

**Figure 2:**
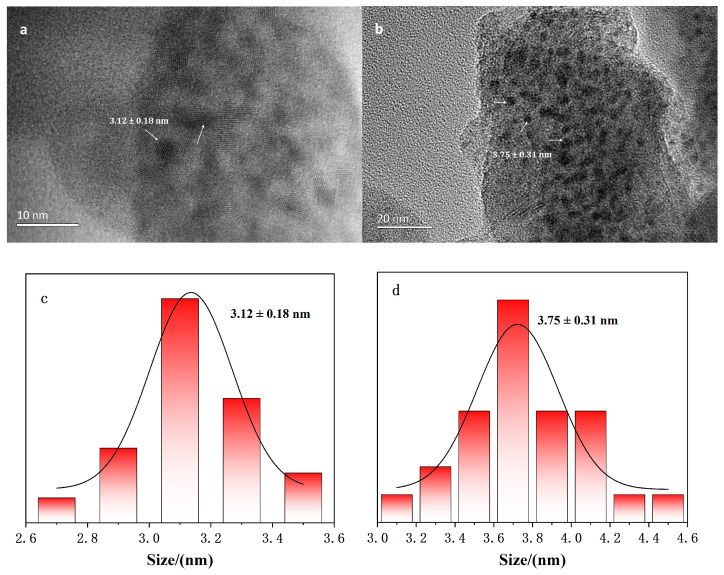
TEM images of (**a**) L-cysteine-modified QDsand (**b**) QD@MIHs, with corresponding size distribution of (**c**) L-cysteine-modified QDs and (**d**) QD@MIHs.

High-resolution transmission electron microscope (TEM) characterization was performed on both L-cysteine-functionalized ZnS quantum dots (QDs) and the QD@MIHs (Figure 2). The prepared nanoparticle diameters were measured from the TEM images using NanoMeasurer 1.2.5, with subsequent statistical analysis performed in OriginPro 2021 (v9.8.0.20) including Gaussian fitting of size distributions. The TEM images (Figure 2a) reveal well-defined lattice structures of the ZnS QDs with uniform particle size distribution, and the average diameter of the QDs is 3.12 ± 0.18 nm. The QD@MIH nanoparticles (Figure 2b) exhibit good dispersity with a moderately increased average diameter of 3.75 ± 0.31 nm. This size expansion results from the successful surface imprinting process, where the molecularly imprinted hydrogel coating endows molecular-recognition functionality to the QDs. The tailored imprinting cavities enable both high-affinity binding and efficient adsorption of homocysteine molecules.

### 2.3. pH-Dependent Effects on QD@MIHs

**Figure 3:**
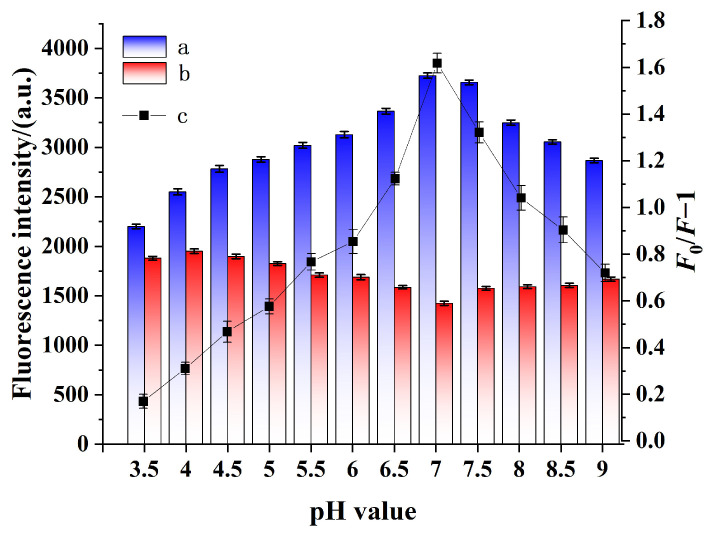
Fluorescence response of QD@MIHs at different pH values (a) without Hcy and (b) with Hcy, (c) Changes in fluorescence quenching efficiency. Experimental conditions: acetic acid–sodium acetate buffer at pH 3.5–5.5; sodium phosphate buffer at pH 6.0–8.0; boric ac-id-borax buffer at pH 8.0–9.0, room temperature.

Figure 3 demonstrates the changes in fluorescence intensity and quenching degree of QD@MIHs before and after Hcy adsorption under different pH conditions. As illustrated in Figure 3, the as-prepared QD@MIHs exhibit obvious pH-dependent fluorescence behavior in the absence of Hcy. Under acidic conditions (pH 3.5–5.0), the fluorescence intensity is relatively low. The emission intensity gradually increases with rising pH values, reaching its maximum at pH 7.0. However, further increasing the pH to alkaline conditions (7.0–9.0) leads to a slight decrease in fluorescence intensity. This phenomenon can be explained by (1) acidic hydrolysis of QD@MIHs generating H_2_S_2_, which quenches fluorescence [43], and (2) alkaline-induced aggregation or precipitation of quantum dots that which affects both material stability and fluorescence efficiency. In contrast, after the addition of Hcy, QD@MIHs exhibited significant fluorescence quenching with increasing pH (3.5–7.0). The maximum fluorescence quenching is achieved at pH 7.0, coinciding with the peak quenching efficiency (*F*_0_/*F* − 1). This phenomenon can be attributed to the pH-responsive properties imparted by the AAm-MAA copolymerized hydrogel layer on the QD surfaces during QD@MIHs fabrication. At pH 7.0, the hydrogel layer swells, facilitating Hcy’s entry into the binding cavities. This enhanced accessibility enables more effective Hcy capture by QD@MIHs, followed by electron transfer between QDs and Hcy that induces pronounced fluorescence quenching. These experimental results demonstrate that pH 7.0 provides maximum quenching efficiency (*F*_0_/*F* − 1) for Hcy detection by QD@MIHs. Therefore, pH 7.0 was selected as the optimal condition for Hcy detection in subsequent experiments.

### 2.4. Rebinding Experiments

#### 2.4.1. Binding Kinetics

**Figure 4:**
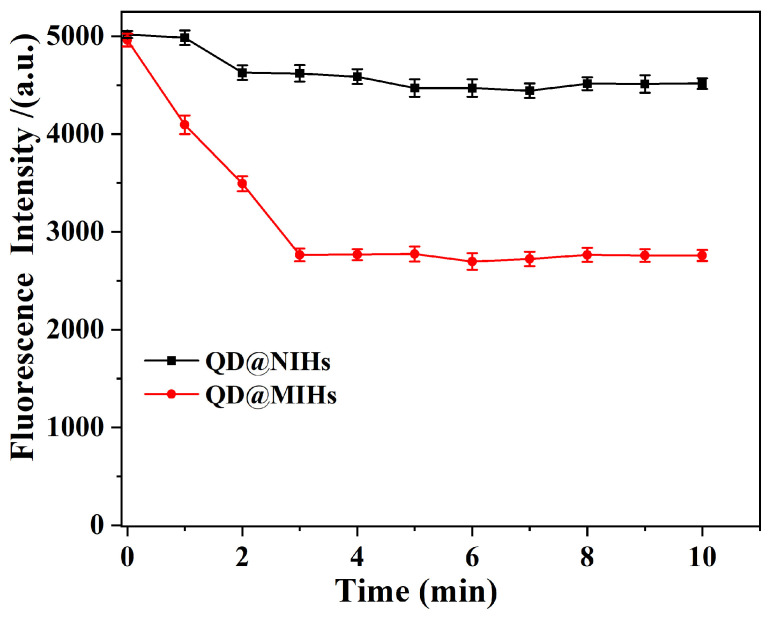
Binding kinetics of QD@MIHs and QD@NIHs in Hcy sample solution.

The adsorption kinetics of the prepared QD@MIHs and QD@NIHs were investigated by incubating them in homocysteine solutions and recording the time-dependent fluorescence intensity variations. As shown in Figure 4, when Hcy was present in the sample, the fluorescence intensity of QD@MIHs decreased rapidly within the first 3 min and then gradually stabilized over the remaining 7 min, ultimately achieving adsorption equilibrium within 5 min. To further investigate the effect of nonspecific adsorption on the prepared materials, the adsorption kinetics of QD@NIHs were measured under same conditions. It can be clearly observed that the fluorescence intensity of QD@NIHs decreased during the initial 2 min, with a slight change occurring in the subsequent 8 min. This phenomenon occurs because of QD@NIHs lacks specific Hcy recognition sites, and the QD@NIHs bind Hcy through nonspecific physical adsorption, resulting in fluorescence quenching. However, due to its limited binding capacity, the system quickly reaches equilibrium, after which the fluorescence intensity of QD@NIHs remains essentially unchanged. In contrast, QD@MIHs feature a molecularly imprinted hydrogel coating that creates tailored recognition cavities during imprinting. These cavities exhibit complementarity to Hcy in term of molecular size, functional group orientation, and three-dimensional structure. Hcy molecules are selectively captured and bound to the QDs surfaces, resulting in pronounced fluorescence quenching and a longer equilibration time. These experimental results demonstrate that the Hcy adsorption equilibrium time of QD@MIHs is 5 min.

#### 2.4.2. Adsorption Isotherm Experiments

**Figure 5:**
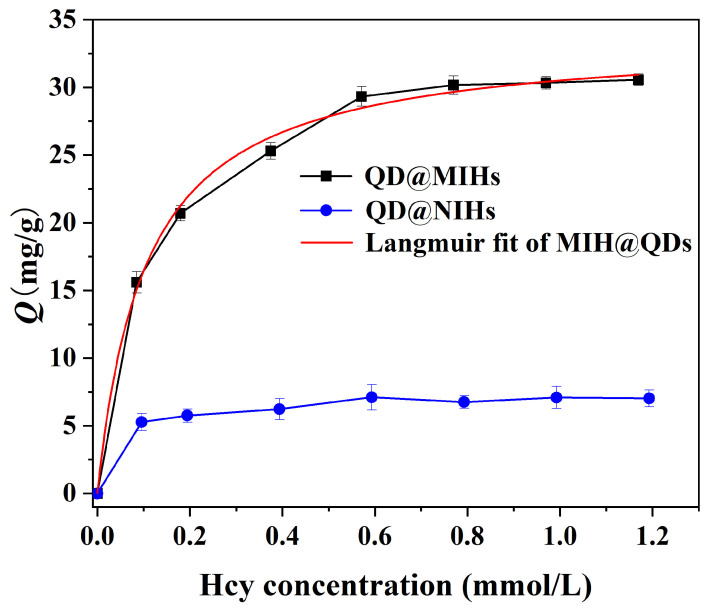
The adsorption isotherm of QD@MIHs and QD@NIHs and Langmuir fit of QD@MIHs.

The adsorption behavior of the prepared quantum dot-based molecularly imprinted hydrogels toward homocysteine was systematically investigated through rebinding experiments. As shown in Figure 5, the Hcy adsorption isotherms of QD@MIHs and QD@NIHs for Hcy were determined. The results demonstrate that the Hcy adsorption capacity of QD@MIHs increased rapidly with increasing Hcy concentrations. Notably, QD@MIHs exhibited significantly enhanced adsorption capacity compared to their non-imprinted counterparts (QD@NIHs). This remarkable performance can be attributed to the precisely constructed three-dimensional imprinted cavities within the molecularly imprinted hydrogel matrix coating the quantum dots, which provide numerous specific binding sites for effective Hcy recognition and capture. The molecular imprinting technology endowed the QD@MIHs with superior molecular recognition capability, thereby achieving substantially improved adsorption capacity for the target molecule (Hcy). The experimental adsorption isotherm was fitted using both Scatchard and Langmuir models. The Scatchard analysis displayed two distinct linear regressions with different slopes. The corresponding fitting equations were *Q*_e_/*C*_e_ = 0.00105 *Q*_e_ − 0.01175 (*R*^2^ = 0.9786) and *Q*_e_/*C*_e_ = 0.03140 *Q*_e_ − 0.9212 (*R*^2^ = 0.9850), respectively. The experimental adsorption isotherm was fitted using the Langmuir equilibrium model, and the fitting results are presented in Figure 5. The Langmuir Hcy adsorption isotherm equation of QD@MIHs was *C*_e_/*Q* = 0.02083 *C*_e_ + 0.02966 (*R*^2^ = 0.9958). The fitting results demonstrate that the adsorption behavior of the molecularly imprinted material conforms well to the Langmuir model (*R*^2^ = 0.9958), indicating a monolayer adsorption process on homogeneous surfaces. This suggests that the adsorption sites are uniformly distributed on the molecularly imprinted hydrogel surface, and the three-dimensional imprinted cavities exhibit excellent spatial complementarity with Hcy molecules.

### 2.5. Fluorescence Sensing of Hcy Using QD@MIHs

**Figure 6:**
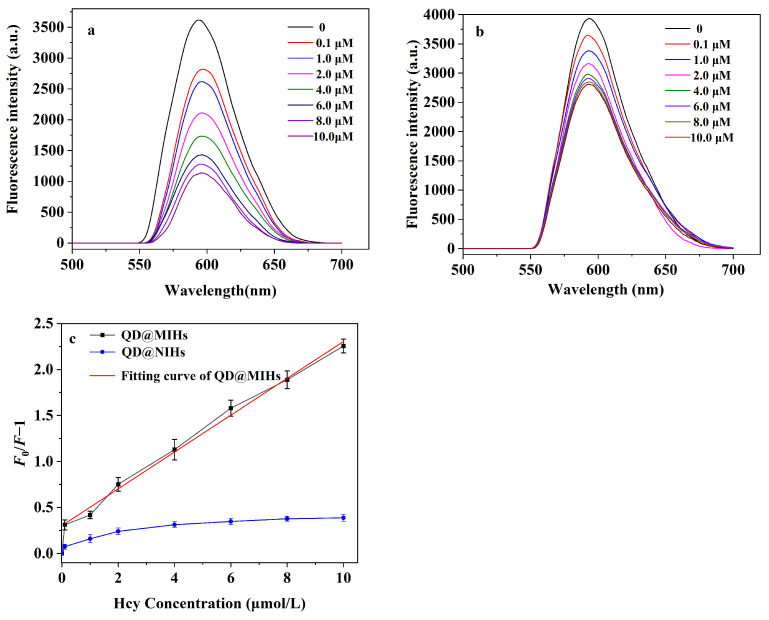
(**a**) Fluorescence quenching spectra of QD@MIHs and (**b**) QD@NIHs at different Hcy concentrations; (**c**) Fitting curve of QD@MIHs fluorescence quenching degree versus Hcy concentration.

Figure 6 shows the changes in the fluorescence emission spectra of QD@MIHs at different Hcy concentrations in the samples. The synthesized QD@MIHs exhibit narrow and symmetric fluorescence emission with a large full width at half maximum (FWHM) and strong fluorescence intensity. After binding with Hcy molecules, the prepared QD@MIHs display significant fluorescence quenching, primarily attributed to the electron transfer process between the quantum dots and the template molecules. As shown in Figure 6, the fluorescence intensity of both QD@MIHs and QD@NIHs gradually decreased as the concentration of Hcy increased. Compared to QD@NIHs, QD@MIHs exhibited significant fluorescence quenching, demonstrating a much higher fluorescence quenching efficiency (*F*_0_/*F* − 1) than QD@NIHs (Figure 6b). This pronounced fluorescence quenching of QD@MIHs can be attributed to the molecular imprinting process employed in QD@MIH synthesis. During imprinting process, non-covalent interactions including hydrogen bonding and van der Waals forces between functional monomers and Hcy molecules generated complementary binding cavities in the molecularly imprinted hydrogel following template removal. These imprinted cavities matched Hcy in term of spatial structure, size, shape, and functional groups, and facilitated more selective binding of Hcy molecules to the molecularly imprinted hydrogel layer on QD@MIHs. Subsequently, the electron transfer between the quantum dots and bound Hcy molecules resulted in the observed fluorescence quenching phenomenon. As a control, the adsorption of Hcy molecules by QD@NIHs mainly occured through nonspecific interactions, resulting in limited Hcy adsorption capacity and a lower degree of fluorescence quenching compared to QD@MIHs. Figure 6a demonstrates that as the Hcy concentration increases in the samples, the fluorescence intensity of QD@MIHs decreases proportionally, indicating that calculating the Hcy concentration in the solution based on fluorescence quenching is feasible and effective. Under optimal conditions, when the Hcy concentration ranges from 0.1 to 10.0 μM, the degree of fluorescence quenching (*F*_0_/*F* − 1) of the QD@MIHs exhibits a good linear relationship with Hcy concentration, with a correlation coefficient of 0.9972. The corresponding method detection limit is 0.027 μM (3 *δ*/*S*). The detection method developed in this study demonstrates convenient operation, rapid detection, cost-effectiveness, and high selectivity.

### 2.6. Selective Experiments

**Figure 7:**
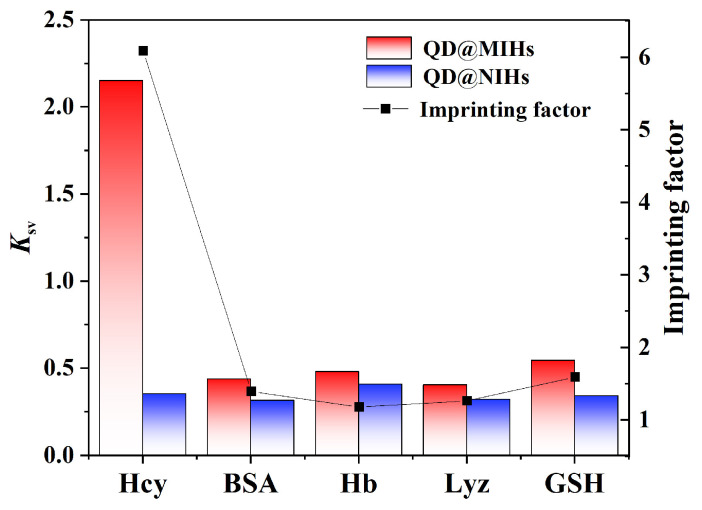
The imprinting factor (IF) and fluorescence quenching constant (*K_sv_*) of QD@MIHs and QD@NIHs at different sample solution concentrations.

To evaluate the specific Hcy recognition capability of QD@MIHs for Hcy, bovine serum albumin (BSA), lysozyme (Lyz), hemoglobin (Hb) and glutathione (GSH) were selected as reference molecules for comparative analysis. As shown in Figure 7, within the concentration range of 0.1–10.0 μM, Hcy exhibited significantly higher fluorescence quenching constants (*Ksv*) for QD@MIHs compared to other interfering molecule (BSA, Lyz, Hb and GSH). This phenomenon can be attributed to the numerous Hcy imprinting sites created on the molecularly imprinted hydrogels during the imprinting process. These binding sites demonstrated complementary interactions with Hcy molecules in terms of spatial configuration, size matching, functional groups and surface charge distribution, thereby enabling enhanced Hcy binding and more pronounced fluorescence quenching of QD@MIHs. On the other hand, the bovine serum albumin, lysozyme, Hb and GSH molecules did not match the imprinted sites on the QD@MIHs surface. Their adsorption primarily relied on non-specific interactions such as physical adsorption, resulting in low adsorption capacity for the target molecules and thus a weaker degree of fluorescence quenching. Furthermore, the quenching effect of the Hcy molecules and reference molecules on the QD@NIHs showed no significant difference, and their quenching constants were quite similar. This is mainly because the QD@NIHs were prepared without corresponding template molecules, resulting in the absence of imprinted sites for the target analyte. The binding between the analyte molecules and QD@NIHs was primarily non-specific, leading to weak adsorption and thus an insignificant quenching effect. Experimental results demonstrated that the imprinting factor for Hcy was 6.08, significantly higher than those of the reference molecules (BSA: 1.39; Lyz: 1.18; Hb: 1.26; GSH: 1.59), indicating that the prepared QD@MIHs had high selectivity for Hcy molecules. The QD@MIHs prepared in this study were compared with other reported Hcy detection techniques, as shown in Table 1. Compared to other Hcy detection methods (such as HPLC, fluorescent probes, and Au NPs), QD@MIHs exhibited a good linear range, a lower detection limit, and faster response times for Hcy detection. The developed method is simple to operate, has low instrumental requirements, and does not require complex sample processing, while also demonstrating good selectivity.

### 2.7. Applications

This experiment adopted the standard addition recovery method to validate the accuracy and reliability of the prepared molecularly imprinted hydrogel for detecting Hcy in urine samples. Since all samples were collected from healthy volunteers with no detectable endogenous Hcy in their urine, Hcy standards at varying concentrations were spiked into the urine samples to validate the accuracy of QD@MIHs detection. The recovery rates are presented in Table 2. As shown in Table 2, the recovery rates of Hcy ranged from 94.34% to 104.1%, with relative standard deviations (RSD, *n* = 3) between 3.56% and 7.17%, demonstrating satisfactory performance. The results indicate that the fabricated quantum dot-based molecularly imprinted hydrogel can effectively detect Hcy in real samples. The method is accurate, reliable, highly sensitive, and suitable for the rapid analysis of Hcy in practical applications.

### 2.8. Reusability and Stability of QD@MIHs

**Figure 8:**
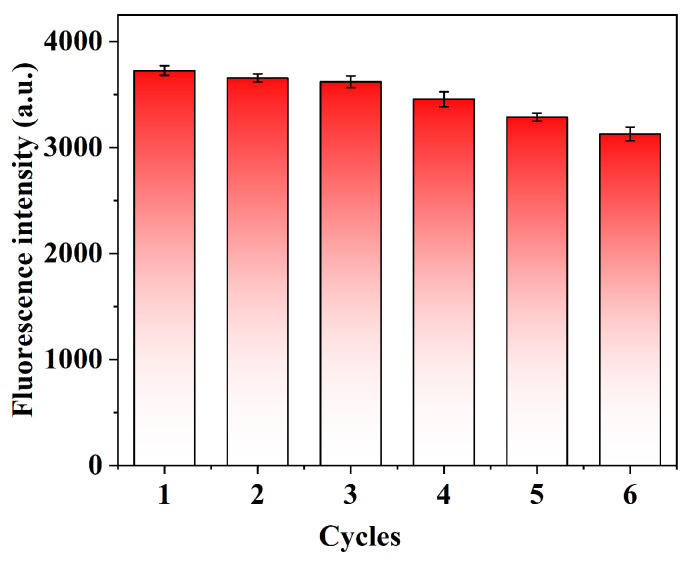
The reusability of QD@MIHs.

**Figure 9:**
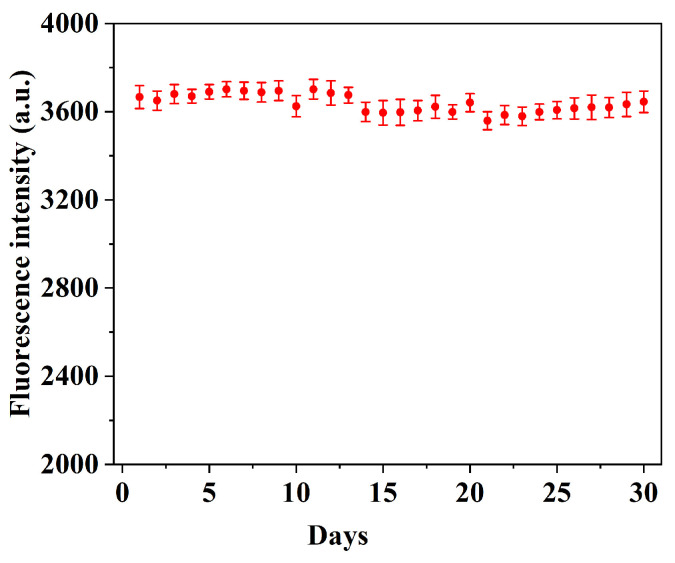
The fluorescence stability of QD@MIHs.

This experiment evaluated the reusability of the prepared QD@MIHs. the changes in fluorescence intensity after five consecutive elution-adsorption cyclesare shown in Figure 8. As depicted in Figure 8, the fluorescence intensity of QD@MIHs showed no significant variation during the first three cycles. After the fourth cycle, the fluorescence intensity decreased by 10.5%. Repeated QD@MIH regeneration cycles lead to gradual ligand loss from the QDs surface and structural damage to the molecularly imprinted hydrogel coating, ultimately resulting in performance decline. These results indicate that the prepared QD@MIHs maintain good reusability within three operational cycles. Additionally, after 30 days of storage at 4 °C in the dark, the QD@MIHs had excellent fluorescence performance with no significant change in their intensity (Figure 9), demonstrating their high fluorescence stability.

## 3. Conclusions

This study successfully developed a water-soluble quantum dot-based molecularly imprinted hydrogel sensor (QD@MIHs) for highly efficient detection of Hcy molecules. As advanced fluorescent biosensors, QD@MIHs integrate the optical properties of quantum dots with the high selectivity of molecularly imprinted hydrogels, significantly enhancing the selectivity and accuracy of Hcy detection in complex samples. The QD@MIHs demonstrates user-friendly operation, a rapid response, and greatly improved detection efficiency. The Experimental results demonstrate that QD@MIHs can effectively identify and detect Hcy in samples with a rapid response time and excellent selectivity. Furthermore, the QD@MIHs exhibit reliable accuracy in real sample analysis, along with good stability and reusability. As fluorescent biosensor, QD@MIHs enable real-time monitoring of Hcy concentrations, showing significant potential for early disease screening and point-of-care diagnostics, with broad applications in preventive medicine and biomedical research.

## 4. Materials and Methods

### 4.1. Instruments and Reagents

Zinc sulfate (ZnSO_4_·7H_2_O), manganese chloride (MnCl_2_·4H_2_O), sodium sulfide (Na_2_S·9H_2_O), ammonium persulfate, L-homocysteine (Hcy), tris (2-carboxyethyl)phosphine hydrochloride (TCEP), L-cysteine, bovine serum albumin (BSA), lysozyme (Lyz), hemoglobin (Hb) and glutathione (GSH) were purchased from Maclin Biochemical Technology Co., Ltd. (Shanghai, China). Acrylamide (AAm), methacrylic acid (MAA), *N,N’*-methylene diacrylamide (MBAAm), tetramethylenediamine (TEMED), and potassium persulfate (KPS) were obtained from Aladdin Biochemical Technology Co., Ltd. (Shanghai, China). Acetic acid and anhydrous ethanol were purchased from Sinopharm Group Chemical Reagent Co., Ltd. (Shanghai, China). All of the reagents were of analytical grade, and the water used in experimental water was ultra-pure water.

The fluorescence spectra were measured by an FL-970 spectrofluorometer (Techcomp, Shanghai, China). The composition of the nanomaterials was analyzed using a JEM F200 field emission transmission electron microscope (JEOL, Tokyo, Japan). The UV/vis adsorption spectra were determined with a Specord 210 plus spectrophotometer (Analytik, Jena, Germany).

### 4.2. Synthesis of L-Cysteine-Modified Quantum Dots

The synthesis of L-cysteine-modified quantum dots was performed via a sequential aqueous-phase reaction [49]. Firstly, zinc sulfate heptahydrate (12.5 mmol) and manganese chloride tetrahydrate (1.0 mmol) were co-dissolved in 90 mL of deionized water. The precursor solution was stirred for 3 h at 25 °C under nitrogen protection. Secondly, a freshly prepared sodium sulfide solution (15.0 mmol in 10 mL H_2_O) was added dropwise (0.5 mL/min) to the reaction mixture, which was maintained at 60 °C for 10 h. Thirdly, L-cysteine (0.5 mmol in 5 mL H_2_O) was introduced to functionalize the nanoparticle surfaces during a 12 h dark-phase reaction. Finally, the resulting colloidal products were purified through three cycles of centrifugation (8000 rpm) and redispersion in deionized water to remove the residual reactants. The purified precipitate was collected and vacuum-dried for subsequent experiments.

### 4.3. Fabrication of Molecularly-Imprinted-Hydrogel-Capped QDs

Surface imprinting technology was employed to coat the homocysteine molecule-imprinted hydrogel onto the L-cysteine-modified QDs, constructing a QD@MIHs fluorescence sensor as illustrated in Figure 1. First, 3.6 mmol AAm, 1.2 mmol MAA, and 1.0 mmol Hcy were sequentially added to 50 mL of pure water and ultrasonically dispersed for 20 min. Next, 200 mg of L-cysteine-modified QDs were added, followed by 5 min of ultrasonic dispersion and the addition of0.18 mmol MBAAm were added. After stirring for 30 min, 15 mg KPS and 8 μL TEMED were added to initiate polymerization. The mixed solution was reacted at room temperature under nitrogen protection for 20 h. As a control, non-imprinted hydrogels (QD@NIHs) were prepared using the same protocol without Hcy molecules. The products were washed with water and ethanol to remove residual reactants. The QD@MIHs were then eluted with an SDS/acetic acid solution (1%: 10%, *w*/*v*) until their fluorescence intensity was the same as that of the QD@NIHs. Finally, the product was vacuum-dried and stored in darkness at 4 °C. The QD yields of the Mn: ZnSQDs, QDs@MIHs, and QDs@NIHs in ethanol which were 38.3%, 22.5%, and 23.7%, respectively.

### 4.4. Effect of pH on the QD@MIHs

In a 10 mL calibrated test tube, 1 mL of 0.1 g/L QD@MIHs and QD@NIHs dispersions were prepared in 9 mL buffer solutions across varying pH ranges (acetic acid–sodium acetate buffer at pH 3.5–5.5; sodium phosphate buffer at pH 6.0–8.0; boric acid–borax buffer at pH 8.0–9.0) and sonicated for 5 min. After 5 min of incubation at 25 °C, the change in fluorescence intensity of QD@MIHs in the presence and absence of Hcy in different buffer solutions was measured using an FL-970 spectrofluorometer.

### 4.5. Adsorption Experiments

#### 4.5.1. Adsorption Kinetics

The adsorption kinetics of the QD@MIHs in Hcy solution were first investigated. A standard Hcy solution was prepared by dissolving 0.135 g of Hcy in 100 mL of phosphate buffer (pH 7.0), with 1.2 mmol TCEP added to prevent oxidative degradation. For the adsorption experiments, QD@MIHs or QD@NIHs were ultrasonically dispersed in 10 μmol/L Hcy solution. The mixtures were incubated at 25 °C with orbital shaking (150 rpm) for varying durations, and the time-dependent fluorescence intensity changes in both the QD@MIHs and QD@NIHs were recorded.

#### 4.5.2. Binding Isotherm Experiments

In the rebinding experiments, QD@MIHs or QD@NIHs were dispersed in 10 mL of phosphate buffer (pH 7.0) containing varying concentrations of Hcy (0–1.2 mmol/L) at 25 °C. Binding isotherm studies were conducted by determining the adsorption capacities of QD@MIHs and QD@NIHs across an Hcy concentration range of 0.1–1.2 mmol/L. The adsorbed protein quantity (Q, mg/g) was calculated using the equation

(1) $$Q=(C_{0}-C_{e})V/W$$

where *C*_0_ and *C_e_* represent the initial and equilibrium concentrations of Hcy, respectively; *V* is the solution volume; and *W* denotes the mass of QD@MIHs or QD@NIHs.

### 4.6. Determination of Homocysteine Using QD@MIHs

At room temperature (25 °C), homocysteine (Hcy) solutions were prepared by dissolving Hcy in phosphate buffer (pH 7.0). TCEP was then added as a reducing agent, followed by nitrogen (N_2_) purging for 15 min to prevent oxidative degradation. A series of Hcy standard solutions with concentrations ranging from 0 to 10.0 μmol/L were prepared. QD@MIHs were sequentially added to these solutions, achieving a final concentration of 10 mg/L in all reaction systems. After thorough mixing, the solutions were incubated on a shaker at room temperature for 5 min. the fluorescence emission spectra of the QD@MIHs were recorded at an excitation wavelength of 310 nm, with QD@NIHs as the control. All experiments were performed in triplicate. The fluorescence quenching of the QD@MIHs followed the Stern–Volmer equation, as expressed below:

(2) F0F=1+KSV[Q]

Here, *F*_0_ represents the initial fluorescence intensity, where *F* denotes the fluorescence intensity with the quencher (Hcy); *K_sv_* is the Stern–Volmer constant; and [*Q*] is the concentration of the quencher (Hcy).

### 4.7. Selective Experiments Methods

In this experiment, we selected BSA, Lyz, Hb, and GSH as competing substances for Hcy to evaluate the selective recognition capability of the prepared QD@MIHs. The choice of competing analytes was based on the following biological rationale: (i) glutathione (GSH) was included as a potential metabolic interferent due to its similar sulfhydryl structure to homocysteine (Hcy); (ii) bovine serum albumin (BSA), the most abundant protein in plasma, was used to simulate the matrix environment; (iii) hemoglobin (Hb) was in-cluded because it is a key interferent released during red blood cell lysis; and (iv) lysozyme (Lyz) was included as it is commonly present in secretions at inflammatory sites. A series of sample solutions with varying concentrations (0–10.0 μmol/L) of Hcy, BSA, lysozyme, Hb, and GSH were prepared. The prepared QD@MIHs and QD@NIHs were then added to these sample solutions to achieve a final QD@MIH concentration of 10 mg/L in all samples. After incubation at room temperature for 5 min, the changes in fluorescence intensity of QD@MIHs and QD@NIHs were measured and recorded. The imprinting factor (IF) was used to evaluate the selectivity of QD@MIHs and QD@NIHs toward each substance, and the calculation formula is as follows:

(3) $$IF=K_{SV,MIP}/K_{SV,NIP}$$

where *K*_sv,MIP_ and *K*_sv,NIP_ are the quenching constants for QD@MIHs and QD@NIHs for different sample solutions, respectively.

### 4.8. Application in Real Samples

To validate the practical application of the fabricated QD@MIHs sensor, urine samples were employed as real samples to evaluate the practical Hcy detection performance of the prepared QD@MIHs. Because urine contains negligible protein-bound Hcy, the developed QD@MIHs-based sensor primarily aims to detect free-form homocysteine. Urine samples collected from healthy volunteers were filtered through 0.4 μm membranes and stored at 4 °C in the dark. For analytical measurements, 100 μL of the prepared QD@MIHs (1.0 g/L) was dispersed into 9 mL of pretreated urine sample (filtered and reduced with 1 mM TCEP). The mixture was adjusted to pH 7.0 using phosphate buffer (NaH_2_PO_4_/Na_2_HPO_4_), and the final volume was increased to 10 mL with ultrapure water. After mixing, the solution was incubated at 25 °C for 5 min under dark conditions. The changes in fluorescence intensity of QD@MIHs were measured using a spectrofluorometer. To further verify the accuracy of the developed method, a standard addition approach was employed by spiking different levels of homocysteine (0.1, 0.5, 1.0 μmol/L) into the samples for recovery rate determination.

### 4.9. Stability and Reusability

The fluorescence stability of the prepared QD@MIHs was evaluated under dark storage conditions. The QD@MIHs were stored at 4 °C, and their fluorescence intensity was measured continuously for 30 days. Additionally, the reusability of QD@MIHs was systematically investigated. The prepared QD@MIHs underwent consecutive adsorption–de-sorption–adsorption cycles, with fluorescence intensity recorded after each cycle to evaluate their reusability. The specific cycling procedure was as follows: The QD@MIHs were incubated in Hcy samples for 5 min followed by fluorescence measurement. Then, bound homocysteine molecules were removed using SDS/acetic acid solution (1: 10, *m*/*v*), and the QD@MIHs were washed with buffer until a neutral pH was achieved for subsequent reuse.

## Figures and Tables

**Table 1:** Comparison of the performance of the QD@MIHs with previously reported methods for the determination of Hcy.

Analysis Technique	Linearity (μM)	LOD	Detection Time (min)	References
HPLC	0.15–22.2	0.037	30.0	[16]
Terahertz spectroscopy	10.0–150.0	10.0	7.0	[44]
Electrochemical biosensor	5.0–150	1.2	10.0	[45]
Two-photon fluorescent probe	0–10	0.018	60.0	[46]
Aptasensor based on aptamer/Au NPs	1–100	1.0	30.0	[47]
Magnetic imprinted polymer	1.0–2.0	0.03	90.0	[48]
QD@MIHs	0.1–10	0.027	5.0	This work

**Table 2:** The results of the experiments on the Hcy recovery rate in real samples.

Sample	Detected (μM)	Added (μM)	Measured (μM)	Recovery Rates (%)	RSD (*n* = 3, %)
urine sample 1	Nd ^1^	0.1	0.0952	95.2	4.25
		0.5	0.5128	102.6	5.67
		1.0	0.9838	98.4	3.56
urine sample 2	nd	0.1	0.0974	97.4	5.72
		0.5	0.5204	104.1	6.19
		1.0	1.015	101.5	4.23
urine sample 3	nd	0.1	0.0943	94.34	7.17
		0.5	0.5178	103.6	5.21
		1.0	1.012	101.2	5.25

^1^ no Hcy detected.

## Data Availability

All the data generated by this research is included in the article.

## Abbreviations

The following abbreviations are used in this manuscript:

QDs	Quantum dot
Hcy	Homocysteine
MIHs	Molecularly imprinted hydrogel
MIT	Molecular imprinting technology
GC	Gas chromatography
HPLC	High-performance liquid chromatography
IF	Imprinting factor
NIHs	Non-imprinted hydrogels
QD@MIHs	Quantum dot (QD)-based molecularly imprinted hydrogels
